# Solitary rectal ulcer syndrome: MRI findings and differentiation from rectal cancer

**DOI:** 10.1186/s13244-025-01979-7

**Published:** 2025-06-16

**Authors:** Peiyi Xie, Xiaoying Lou, Shuai Fu, Xiaohui Di, Qitong Huang, Zhiming Zeng, Kexin Niu, Junying Zhu, Meiyu Hu, Xiaochun Meng

**Affiliations:** 1 Department of Radiology, The Sixth Affiliated Hospital, Sun Yat-sen University, Guangzhou, China; 2 Biomedical Innovation Center, The Sixth Affiliated Hospital, Sun Yat-sen University, Guangzhou, China; 3 Department of Pathology, The Sixth Affiliated Hospital, Sun Yat-sen University, Guangzhou, China

**Keywords:** Rectal diseases, Ulcer, Rectal cancer, Magnetic resonance imaging

## Abstract

**Background:**

Systematic MRI findings of solitary rectal ulcer syndrome (SRUS) are lacking. We aimed to evaluate the MRI findings of SRUS and to identify the MRI features that differentiate SRUS from rectal cancer.

**Methods:**

This retrospective study consecutively included 30 patients diagnosed with SRUS from January 2015 to December 2021. The clinical and MRI findings of SRUS patients were summarized. We randomly selected 120 rectal cancer patients with ≤ T2N0 pathological staging in a 1:4 ratio of SRUS to rectal cancer cases to perform differential diagnosis analysis.

**Results:**

SRUS patients were significantly younger (mean age ± standard deviation [SD], 37 years ± 17; 22 men) than rectal cancer patients (mean age ± SD, 62 years ± 12; 67 men; *p* < 0.001). Compared to rectal cancer patients, SRUS patients had a significantly higher incidence of ulceration (63.33%), submucosal edema (36.67%), unrestricted diffusion (76.67%), hypo- or high-low mixed intensity on T2-weighted imaging (T2WI, 76.67%), and layer enhancement (40%) (all *p* < 0.001). Interestingly, in the combinations of MRI features including unrestricted diffusion, hypo- or high-low mixed intensity on T2WI, and layer enhancement or submucosal edema showed an excellent diagnostic performance with area under the curve, sensitivity, specificity, positive predictive value, negative predictive value, and accuracy of 0.97 (95% CI: 0.92, 1.00), 93%, 100%, 100%, 98%, and 99%, respectively, in differentiating SRUS from rectal cancer.

**Conclusion:**

The combinations of three MRI features are simple and show excellent diagnostic performance. These may be useful tools for differentiating SRUS from rectal cancer.

**Critical relevance statement:**

The combinations of three MRI features including unrestricted diffusion, hypo- or high-low mixed intensity on T2WI, and layer enhancement or submucosal edema show excellent diagnostic performance, which have potential to serve as useful tools for differentiating SRUS from rectal cancer.

**Key Points:**

- MRI could differentiate solitary rectal ulcer syndrome (SRUS) from rectal cancer.
- SRUS patients had a significantly higher incidence of several MRI features.
- The combinations have potential for differentiating SRUS from rectal cancer.

**Graphical Abstract:**

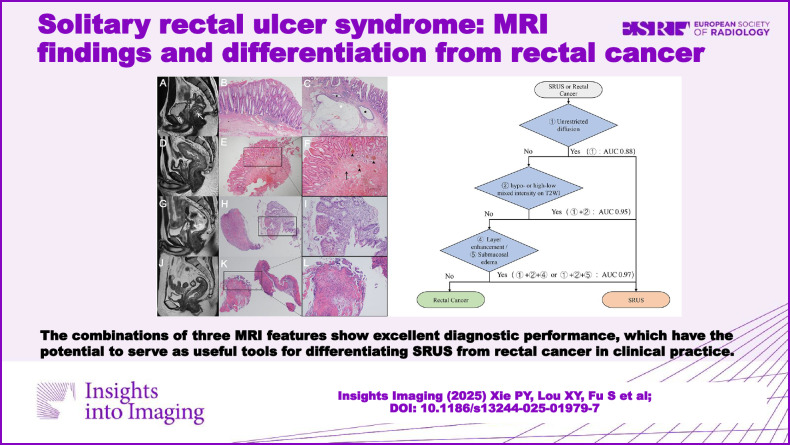

## Introduction

Solitary rectal ulcer syndrome (SRUS) is a rare benign disease characterized by chronic and nonspecific inflammatory ulcers of the rectum. However, its low incidence (only 1 in 100,000 per year) has led to a lack of awareness and recognition among medical professionals [1,2]. While the cause of SRUS remains unclear, several factors have been suggested, including rectal prolapse [2, 3] and pelvic floor dysfunction leading to severe chronic constipation [4]. These factors contribute to a variety of symptoms, including rectal bleeding, straining during defecation, constipation, abdominal pain, and a sense of incomplete evacuation [2–4]. Unfortunately, the diagnosis of SRUS can be challenging due to the nonspecific clinical presentation and endoscopic findings [3]. Differential diagnoses include inflammatory bowel disease (e.g., ulcerative colitis, Crohn’s disease), juvenile polyps, ischemic colitis, and vascular ectasia [5, 6]. Some cases are even misdiagnosed as rectal cancer due to polypoid or mass-like endoscopic appearances [7, 8], leading to inappropriate treatment and unnecessary surgery. Therefore, an accurate diagnosis of SRUS is critical to provide appropriate treatment options.

Due to its excellent soft tissue resolution, magnetic resonance imaging (MRI) is the preferred choice for the diagnosis and differentiation of various rectal diseases. Although there have been several publications [1, 2, 4] reporting the MRI findings of SRUS and its differentiation from rectal cancer, such as cystic lesions in the submucosa and intact muscularis propria in SRUS, which should be compared with early-stage rectal cancer. Unfortunately, the literature to date consists mainly of case reports (listed in Table S1) with limited systematic evaluation of MRI findings. Furthermore, the accurate diagnosis of SRUS remains challenging, mainly due to the lack of understanding of MRI findings, especially the differences compared to rectal cancer.

Therefore, the primary objective of this study was to evaluate the MRI findings of SRUS and to identify the MRI features that differentiate SRUS from rectal cancer.

## Materials and methods

This retrospective study was approved by the Institutional Ethics Committee of the Sixth Affiliated Hospital of Sun Yat-sen University (approval number 2021ZSLYEC-316). Due to the retrospective nature of the study, informed consent was not required.

### Patient studies

**Fig. 1:**
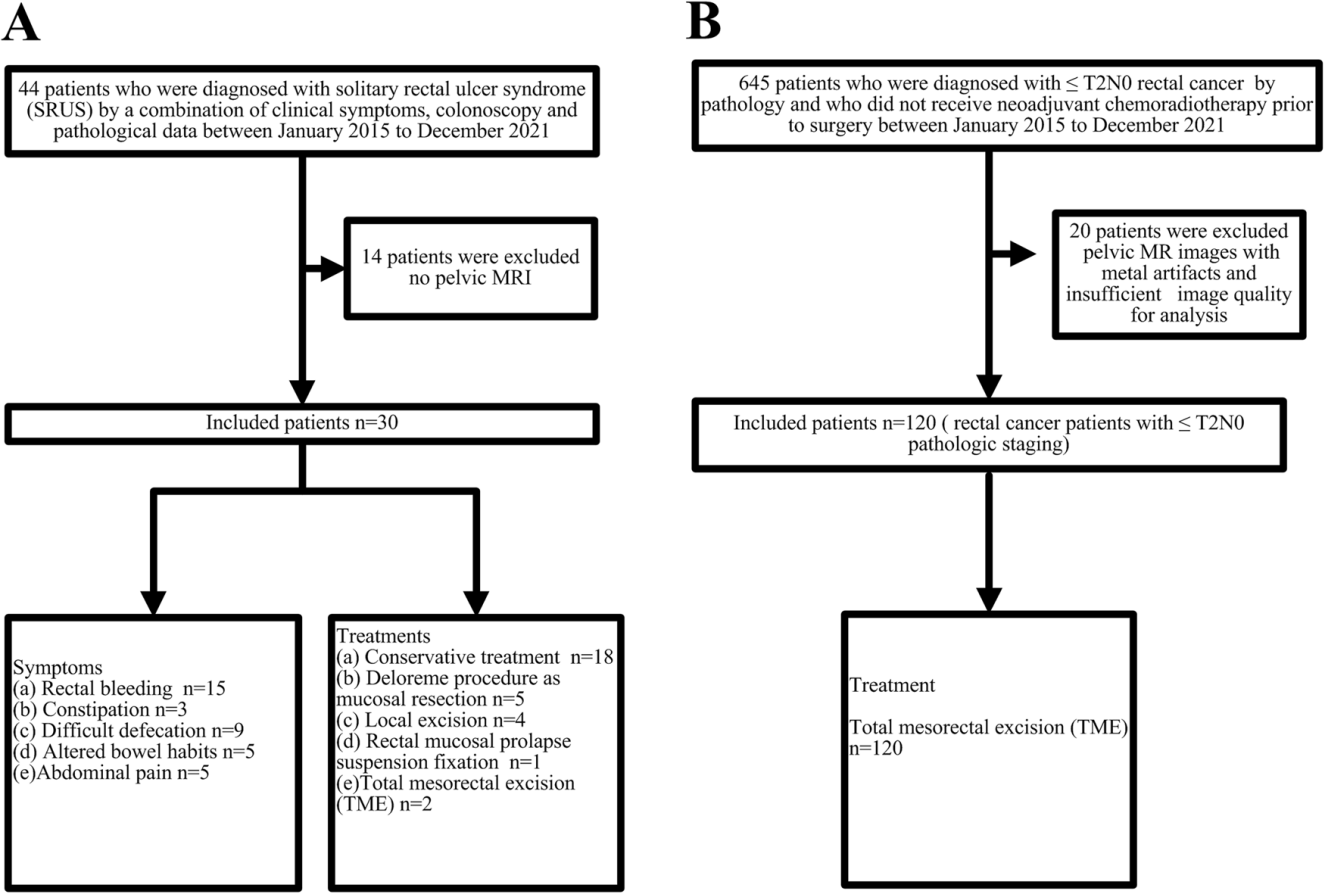
Flowchart summarizing the SRUS (**A**) and rectal cancer (**B**) patients included and excluded in this study

In this retrospective study, with reference to previous literature [1, 2], we consecutively collected data from 44 patients who were diagnosed with solitary rectal ulcer syndrome (SRUS) by a combination of clinical symptoms, colonoscopy and pathological data at the Sixth Affiliated Hospital of Sun Yat-sen University between January 2015 and December 2021. After excluding 14 patients who did not undergo pelvic MRI, a total of 30 eligible patients with SRUS who underwent pelvic MRI were included in this study (details as shown in Fig. 1).

To further improve our understanding of the discriminative features between SRUS and rectal cancer, we performed a comparative analysis. Between January 2015 and December 2021, 645 patients with ≤ T2N0 rectal cancer by pathology who did not receive neoadjuvant chemoradiotherapy before surgery were consecutively selected at the Sixth Affiliated Hospital of Sun Yat-sen University. 120 rectal cancer patients with ≤ T2N0 pathological staging were randomly enrolled in a 1:4 ratio of SRUS to rectal cancer cases (details as shown in Fig. 1). Clinical data, tumor markers (carcinoembryonic antigen, CEA, and carbohydrate antigen 19-9, CA19-9) and MRI findings were analyzed.

### MR imaging

All patients enrolled in this study underwent MR scans with a 3.0-T MR imager (Discovery MR 750w, GE Healthcare), a 1.5-T MR imager (Optimal 360, GE Healthcare), or a uMR S70 1.5-T MR imager (United Imaging Healthcare). The MR studies performed in this study used a standardized high-resolution rectal cancer staging protocol. Scan sequences included high-resolution T2-weighted imaging (T2WI) in the axial, sagittal, and coronal planes, diffusion-weighted imaging (DWI) (b-values of 0 and 800 or 1200 s/mm^2^), and enhanced T1-weighted imaging (T1WI) LAVA sequences (GE) or t1_quick3d_tra_ fs sequences (uMR) with arterial, venous, and delayed-phase acquisitions (details are shown in Table S2). Gadopentine meglumine (Gd-DTPA, Bayer Pharmaceuticals), an enhanced scanning contrast agent, was administered at a dose of 0.2 mmol/kg and a flow rate of 3.0 mL/s.

### Image analysis

Two experienced abdominal imaging radiologists, J.Y. Zhu (with 8 years of clinical experience) and P.Y. Xie (with 10 years of clinical experience), independently reviewed all imaging data from 150 patients. In cases of inconsistency or disagreement, a third highly experienced abdominal imaging radiologist, X.C. Meng (with over 25 years of clinical experience), was consulted to reach a consensus. All were blinded to the clinical data and histological information.

The following parameters were recorded on the MRI images for each patient (as shown in Table 1): (1) ulceration: an ulcer refers to a break or injury in the mucosa [9]; (2) submucosal edema: this sign is characterized by high signal intensity on T2WI, indicating edema in the submucosal layer [10]; (3) cystic lesions in the submucosa: the presence of massive mucus accumulation corresponds to single or multiple submucosal cystic lesions [2]; (4) enlarged mesorectal lymph nodes: lymph nodes with a short-axis diameter greater than 5 mm are considered enlarged [11]; (5) layered enhancement: this feature is identified by hyperenhancement of the inner layer (mucosal layer), iso-enhancement of the outer layer (serosa), and no enhancement of the middle layer (submucosa and muscularis) [12]; (6) enhancement pattern: wash-in and wash-out and persistent enhancement. To analyze the pattern of enhancement, the degree of a lesion was compared with that of adjacent intestinal mucosa; (7) restricted diffusion: refers to hyperintensity in diffusion-weighted imaging (DWI) and hypointensity in apparent diffusion coefficient (ADC) map; (8) intensity on T2WI (slightly hyperintensity, hypointensity, or high-low mixed intensity); (9) muscularis propria intact [2].

**Table 1:** Definition and images of MRI findings

MRI findings	Definitions	Images	
		0 (absent)	1 (present)
Ulceration	An ulcer refers to a break or injury in the mucosa.		
Submucosal edema	This sign is characterized by high signal intensity on T2-weighted images, indicating edema in the submucosal layer.		
Cystic lesions in the submucosa	The presence of massive mucus accumulation corresponds to single or multiple submucosal cystic lesions.		
Enlarged mesorectal lymph nodes	Lymph nodes with a short-axis diameter greater than 5 mm are considered enlarged.		
Layer enhancement	This feature is identified by hyperenhancement of the inner layer (mucosal layer), iso-enhancement of the outer layer (serosa), and no enhancement of the middle layer (submucosa and muscularis).		
Restricted diffusion	Restricted diffusion refers to hyperintensity in DWI and hypointensity in ADC map.		
			
	Hypointensity	Slightly hyperintensity	High-low mixed intensity
T2WI			

Note: These images of A, B and C, D in table 1 are from the patients in Figs 2, 3 and 4, respectively

### Histopathologic analysis

All tissue samples (12 surgical pathology and 18 biopsy pathology of SRUS and 120 surgical pathology of rectal cancer) were stained with H&E and analyzed by a highly experienced pathologist (Xiaoying Lou, with 10 years of experience in colorectal pathology) without prior knowledge of the patients’ clinical and imaging data.

### Statistical analysis

IBM SPSS statistics software, version 25.0 (SPSS Inc.), and R software, version 4.4.1, were used for statistical analysis. Continuous variables were presented as mean ± standard deviation (SD) for normally distributed data or median and interquartile range (IQR) for non-normally distributed data, whereas categorical variables were summarized as counts and percentages for each category. Normal distribution of continuous variables was assessed using the Kolmogorov–Smirnov test, and homogeneity of variance was assessed using Levine’s test. Independent *t*-tests or one-way analysis of variance (ANOVA) were used for normally distributed continuous variables. Non-normally distributed continuous variables were analyzed using Mann–Whitney *U* tests or Kruskal–Wallis tests. Categorical variables were analyzed by chi-squared test or Fisher’s exact test. Interobserver agreement for image quality was assessed using Cohen’s kappa (κ) test. A κ value of 0–0.2 was considered as poor agreement, 0.21–0.4 as fair agreement, 0.41–0.6 as moderate agreement, 0.61–0.8 as good agreement, and 0.81–1.00 as excellent agreement [13]. Diagnostic performance was evaluated by calculating the area under the receiver operating characteristic curve (AUC), sensitivity, specificity, positive predictive value (PPV), negative predictive value (NPV) and accuracy. All tests were two-tailed, and results were considered significant at *p* < 0.05.

## Results

### Clinical characteristics

SRUS patients were significantly younger (mean age ± standard deviation [SD], 37 years ± 17; 22 men) than rectal cancer patients (mean age ± standard deviation [SD], 62 years ± 12; 67 men) (*p* < 0.001). Compared to rectal cancer patients, SRUS patients had a lower rate of CEA levels (*p* = 0.003) and a longer history of symptoms (*p* < 0.001) (see Table 2). Six patients in our study (6/30, 20%) were initially diagnosed with rectal cancer on MRI, but were ultimately diagnosed with SRUS. What’s more, of the 30 SRUS patients, one-third had a good therapeutic response with conservative treatment, one-third had recurrent symptoms after six months of conservative treatment, and one-third initially received conservative treatment but eventually underwent surgery due to recurrent symptoms. All 120 rectal cancer patients underwent total mesorectal excision surgery.

**Table 2:** Clinical characteristics

Variables	SRUS (*N* = 30) %	Rectal cancer (*N* = 120) %	*p*-value
Age (years)	36.77 ± 16.65	61.93 ± 11.53	< 0.001
Gender			0.124
Male	22 (73.33%)	67 (55.83%)	
Female	8 (26.67%)	53 (44.17%)	
CEA (ng/mL)	1.73 (1.55, 2.76)	2.65 (1.73, 3.85)	0.003
CA199 (ng/mL)	4.59 (2.06, 7.58)	5.61 (3.03, 11.86)	0.057
Mean duration of symptoms (months)	12.00 (6.00, 57.00)	3.00 (1.00, 6.00)	< 0.001

Data are means ± standard deviations or numbers. Data in parentheses are ranges or percentages. For the CEA, CA199, data were missing in four patients

### MRI findings of SRUS

Among the 30 SRUS patients, the interobserver agreement for MRI features was excellent for submucosal edema (κ, 0.85; *p* < 0.001), cystic lesions in the submucosa (κ, 1.00; *p* < 0.001), enlarged mesorectal lymph nodes (κ, 0.89; *p* < 0.001), layer enhancement (κ, 0.93; *p* < 0.001), enhancement pattern (κ, 0.87; *p* < 0.001), intensity on T2WI (κ, 0.85; *p* < 0.001) and muscularis propria intact (κ, 1.00; *p* < 0.001) and good for ulceration (κ, 0.62; *p* < 0.001) and restricted diffusion (κ, 0.79; *p* < 0.001) (as shown in Table S3).

**Fig. 2:**
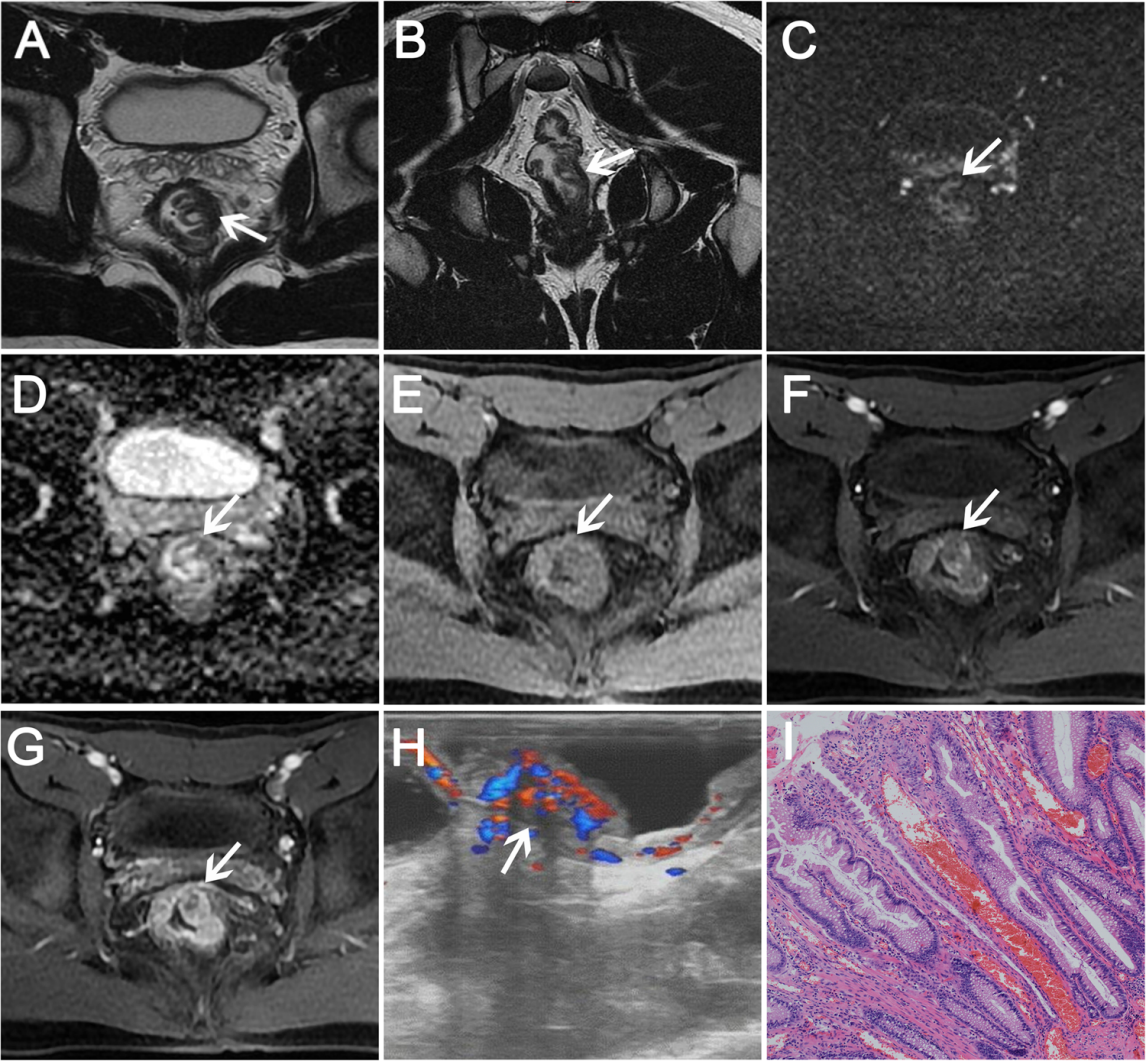
A 22-year-old man with a 1-year history of recurrent rectal bleeding. **A** Axial and (**B**) coronal T2WI show focal thickening and high signal cystic lesions involving a bowel length of approximately 31 mm. **C** Unrestricted diffusion was observed on DWI and (**D**) ADC map. **E** T1WI shows isointense signal in the lesion. **F**, **G** Persistent enhancement with unenhanced cystic component was observed after contrast injection. **H** Endorectal ultrasound showed localized thickening of the rectal wall, mainly mucosal and submucosal thickening, and the muscular layer was still intact. **I** Pathology showed fibromuscular obliteration of the lamina propria, mucinous cell proliferation, and serrated mucosa propria (hematoxylin-eosin stain, magnification, × 100)

**Fig. 3:**
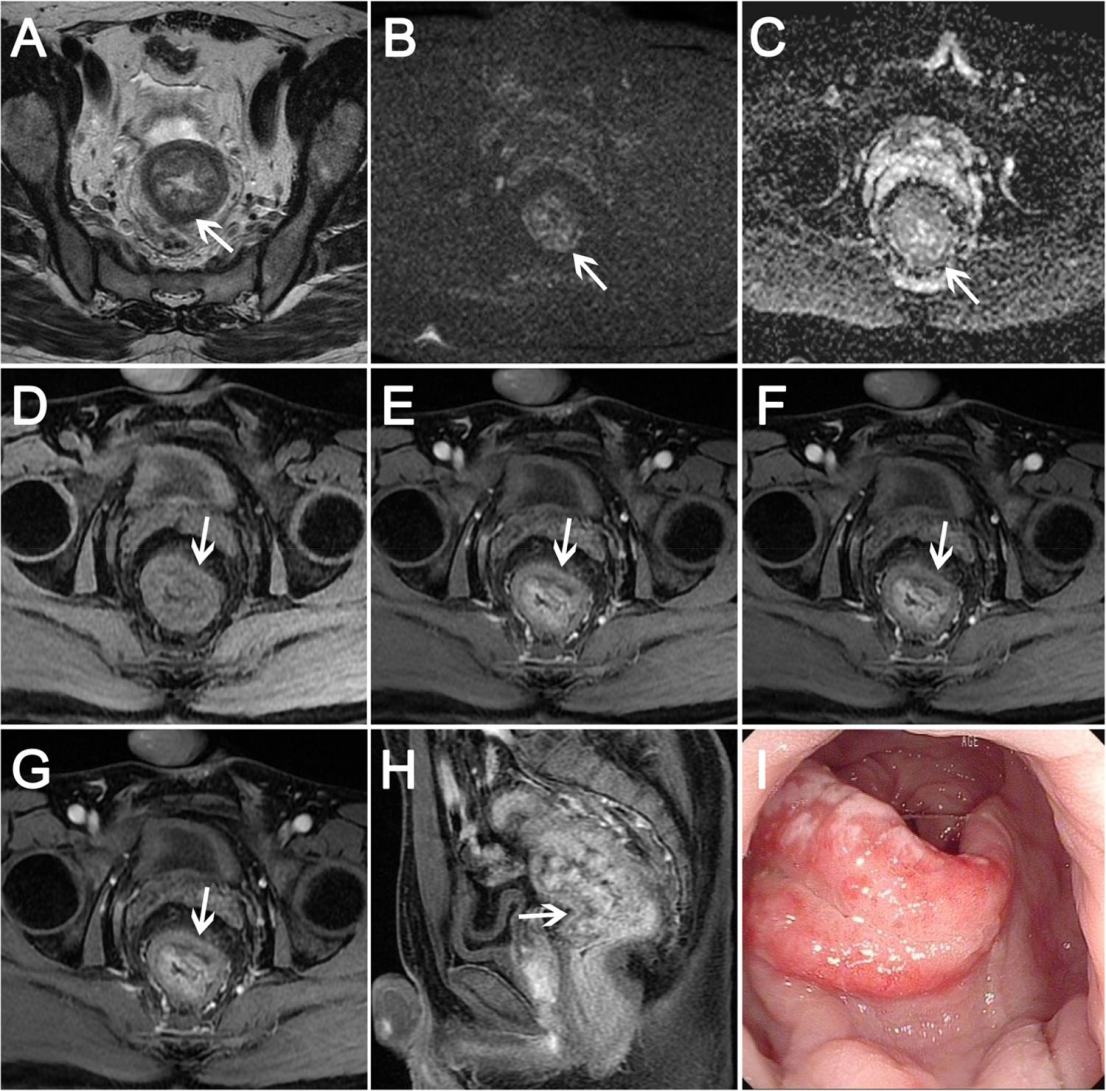
A 35-year-old man presenting with repeated massive prolapse for more than 2 years. **A** Axial T2WI shows diffuse thickening with slightly hyperintensity. **B** Unrestricted diffusion was observed on DWI and (**C**) ADC map. **D** T1WI shows isointense signal. **E**–**H** Persistent and layer enhancement was observed on the multi-phase contrast-enhanced sequences. **I** Colonoscopy shows circumferential irregular mucosal hyperemia, edema, and surface erosion at 30–160 mm from the anal verge

**Fig. 4:**
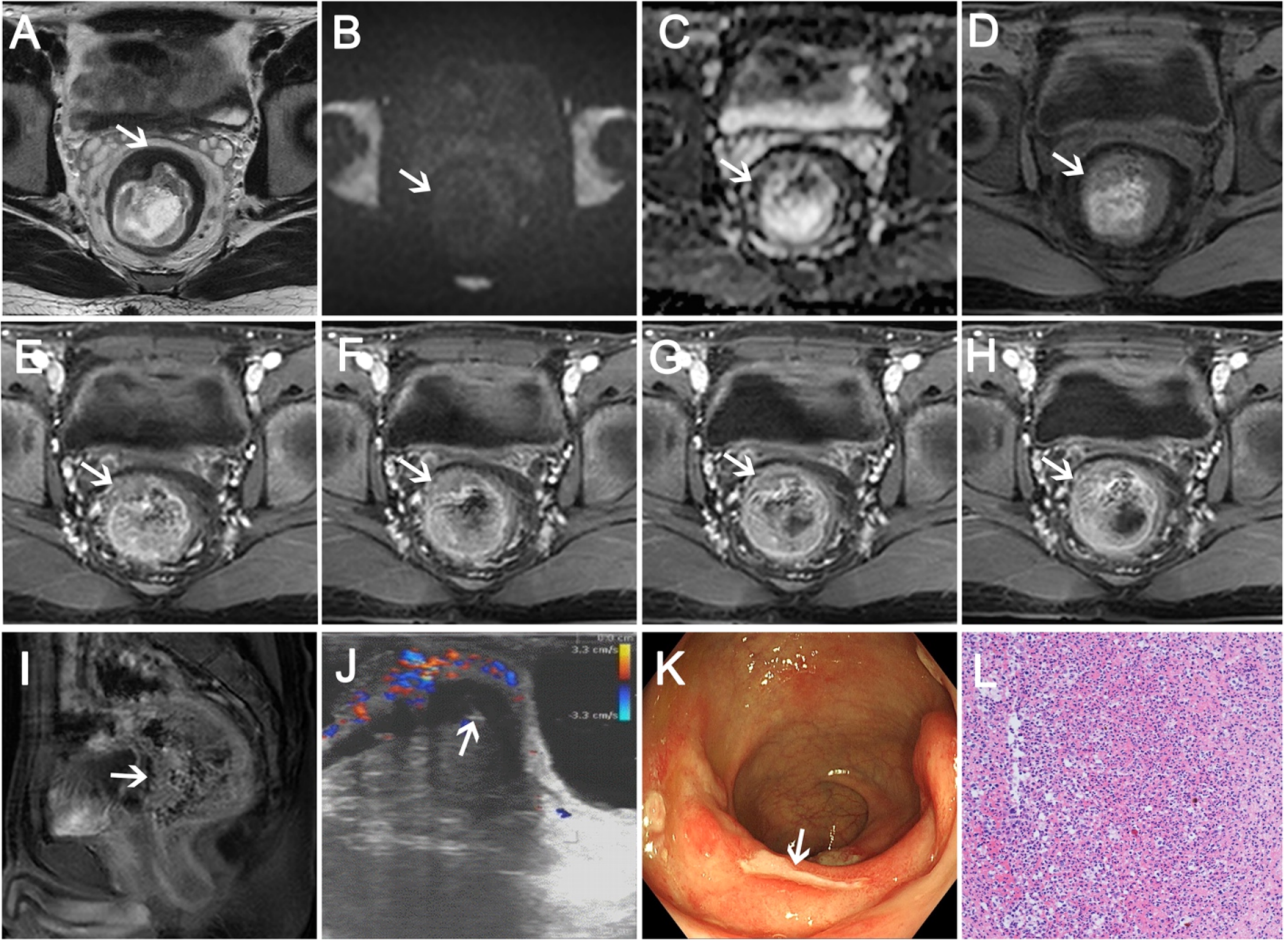
A 34-year-old man with a 2-month history of rectal bleeding. **A** Axial T2WI shows focal thickening with ulceration in the anterior wall of the middle rectum, and this hypointense signal is approximately 26 mm in length. **B** DWI and (**C**) ADC map show unrestricted diffusion. **D** T1WI shows isointense signal. **E**–**I** Persistent enhancement was observed. **J** Endorectal ultrasound shows thickening of the submucosal layer and muscularis propria but intact. **K** Colonoscopy showed a wide rectal lesion with an irregular ulcer. **L** Pathology showed surface ulceration, hematoxylin-eosin stain, magnification, ×100

In 30 SRUS patients, high-low mixed intensity (Figs. 2A, B, S2A, B), hypointensity (Figs. 4A, S1A, B), and slightly hyperintensity (Fig. 3A) were seen on T2WI compared to the muscle signal, and high-low mixed intensity is the most common seen in 16 patients (16/30, 53.34%). 11 patients (11/30, 36.67%) showed submucosal edema (Figs. 3A, S2A, 2B). All patients had an intact muscularis propria. Seven patients (7/30, 23.33%) showed restricted diffusion and 23 patients (23/30, 76.67%) showed unrestricted diffusion (Figs. 2C, D, 3B, C, 4B, C, S1C, D, S2D, E). After enhancement, 12 patients (12/30, 40.00%) showed layer enhancement (Figs. 3D–H, S2C, F–J). Five patients (5/30, 16.67%) showed wash-in/wash-out enhancement, and 25 patients (25/30, 83.33%) showed persistent enhancement (Figs. 2E–G, 3D–H, 4D–I, S1E–H, S2F–J). Ulcerations were found in 19 patients (19/30, 63.33%) (Fig. 4A). Cystic lesions in the submucosa were observed in one patient (1/30, 3.33%) (Fig. 2A, B), and four patients (4/30, 13.33%) showed enlarged mesorectal lymph nodes (Fig. S2D–I).

**Fig. 5:**
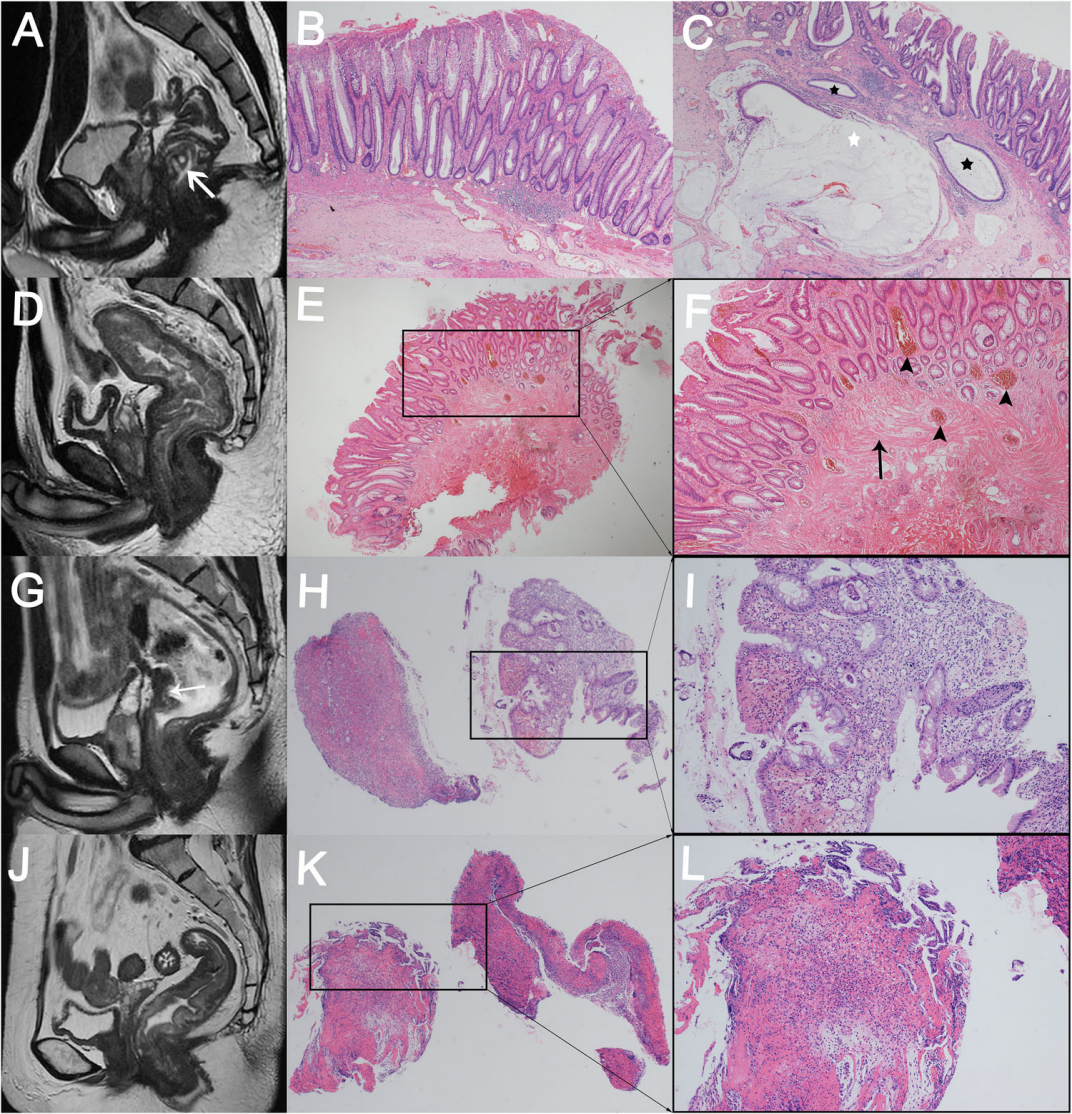
The pathologic basis of MRI findings of SRUS. **A** Sagittal T2WI shows focal thickening and high signal cystic lesions (white arrow) involving a bowel length of approximately 31 mm. **B**, **C** The pathological manifestations showed ectopic crypt glands in submucosa and focal mucus formation (white stars in **C**) with crypt hyperplasia and mucosal erosion **B** (**B**, HE, magnification, × 20; **C**, HE, magnification, × 100). **D** Sagittal T2WI shows diffuse thickening with slightly hyperintensity, involving a bowel length of 164 mm. **E**, **F** Pathologic manifestations included fibromuscular obliteration of the lamina propria, and marked thickened muscularis mucosae (**E**) and crypt hyperplasia and vascular dilation and congestion (**F**) (**E**, HE, magnification, × 20; **F**, HE, magnification, × 100). **G** Focal thickening and ulceration of the anterior wall of the middle rectum on MRI sagittal T2WI, involving a bowel length of 26 mm. **H**, **I** Pathology confirmed ulceration, glandular hyperplasia, and no evidence of malignancy (**H**, HE, magnification, × 20; **I**, HE, magnification, × 100). Diffuse thickening and multiple ulcerations of the middle and upper rectal segments on sagittal T2WI (**J**), involving a bowel length of 76 mm. **K**, **L** Pathology showed surface ulceration, distorted mucosal architecture, and absence of malignant features (**K**, HE, magnification, × 20; **L**, HE, magnification, × 100) Note: These images in Figs. 5A, D, G, J are from the patients in Figs. 2–4 and Supplement Fig. 2, respectively

We analyzed the pathologic basis of the MRI findings of SRUS (Fig. 5). MRI findings of cystic lesions in the submucosa (Fig. 5A), the pathological manifestations showed ectopic crypt glands in submucosa and focal mucus formation (Fig. 5C) with crypt hyperplasia and mucosal erosion (Fig. 5B), which was easily misdiagnosed as adenocarcinoma. Sagittal T2WI showed diffuse bowel wall thickening and submucosal edema (Fig. 5D). Pathologic manifestations included fibromuscular obliteration of the lamina propria and marked thickened muscularis mucosae (Fig. 5E), which was consistent with the diffuse bowel wall thickening in T2WI. There were also crypt hyperplasia and vascular dilation and congestion (Fig. 5F). There were focal thickening and ulceration of the anterior wall of the middle rectum on sagittal T2WI (Fig. 5G) and diffuse thickening and multiple ulcerations of the middle and upper rectal segments on sagittal T2WI (Fig. 5J). Pathology showed ulceration, glandular hyperplasia, and without malignant features (Fig. 5H, I, K, L).

### Different MRI features of SRUS and rectal cancer

In this study, SRUS patients have a significantly higher incidence of ulceration (19/30, 63.33%) and submucosal edema (11/30, 36.67%) compared to rectal cancer (6/120, 5.00%; 0/120, 0.00%, respectively) (all *p* < 0.001). Interestingly, the three MRI features of unrestricted diffusion (23/30, 76.67% vs. 0/120, 0.00%), hypo- or high-low mixed intensity on T2WI (23/30, 76.67% vs. 0/120, 0.00%), and layer enhancement (12/30, 40.00% vs. 0/120, 0.00%) are significantly higher in SRUS patients compared to rectal cancer patients (all *p* < 0.001). In addition, the length of the bowel involved in SRUS patients (median, 51.00 mm, range, 31.75–107.75 mm) is significantly longer than in rectal cancer patients (median, 20.00 mm, range, 12.75–26.50 mm) (*p* < 0.001) (see Table 3 for details). Representative MRI features are shown in Figs. 2–5 and S1–3.

**Table 3:** Comparison of MRI findings between SRUS and rectal cancer groups

Variables	SRUS (*N* = 30) %	Rectal cancer (*N* = 120) %	*p*-value
Ulceration			**< 0.001**
Present	19 (63.33%)	6 (5.00%)	
Absent	11 (36.67%)	114 (95.00%)	
Submucosal edema			**< 0.001**
Present	11 (36.67%)	0 (0.00%)	
Absent	19 (63.33%)	120 (100.00%)	
Cystic lesions in the submucosa			0.452
Present	1 (3.33%)	0 (0.00%)	
Absent	29 (96.67%)	120 (100.00%)	
Enlarged mesorectal lymph nodes			0.084
Present	4 (13.33%)	4 (3.33%)	
Absent	26 (86.67%)	116 (96.67%)	
Layer enhancement			**< 0.001**
Present	12 (40.00%)	0 (0.00%)	
Absent	18 (60.00%)	120 (100.00%)	
Pattern of enhancement			**0.029**
Wash in/wash out	5 (16.67%)	48 (40.00%)	
Persistent enhanced	25 (83.33%)	72 (60.00%)	
Restricted diffusion			**< 0.001**
Present	7 (23.33%)	120 (100%)	
Absent	23 (76.67%)	0 (0.00%)	
Intensity on T2WI			**< 0.001**
Slightly hyperintensity	7 (23.33%)	120 (100.00%)	
Hypointensity	7 (23.33%)	0 (0.00%)	
High-low mixed intensity	16 (53.34%)	0 (0.00%)	
Muscularis propria intact			1.000
Present	30 (100.00%)	120 (0.00%)	
Absent	0 (0.00%)	0 (0.00%)	
Length of involved bowel (mm)	51.00 (31.75, 107.75)	20.00 (12.75, 26.50)	**< 0.001**
Primary lesion location			0.054
Upper rectum	2 (6.67%)	17 (14.17%)	
Middle rectum	13 (43.33%)	70 (58.33%)	
Low rectum	15 (50.00%)	33 (27.50%)	
The lesion involves the circumference of the bowel			0.199
Anterior	5 (16.67%)	27 (22.50%)	
Posterior	3 (10.00%)	22 (18.33%)	
Left lateral	5 (16.67%)	26 (21.67%)	
Right lateral	7 (23.33%)	27 (22.50%)	
Circumferential	10 (33.33%)	18 (15.00%)	

Data are number of participants, and data in parentheses are percentages; mean data are ± SD

Bold values indicate the statistically significant difference between the SRUS and rectal cancer groups, *p* < 0.05

### Performance of the combinations of three MRI features for differentiating SRUS from rectal cancer

**Fig. 6:**
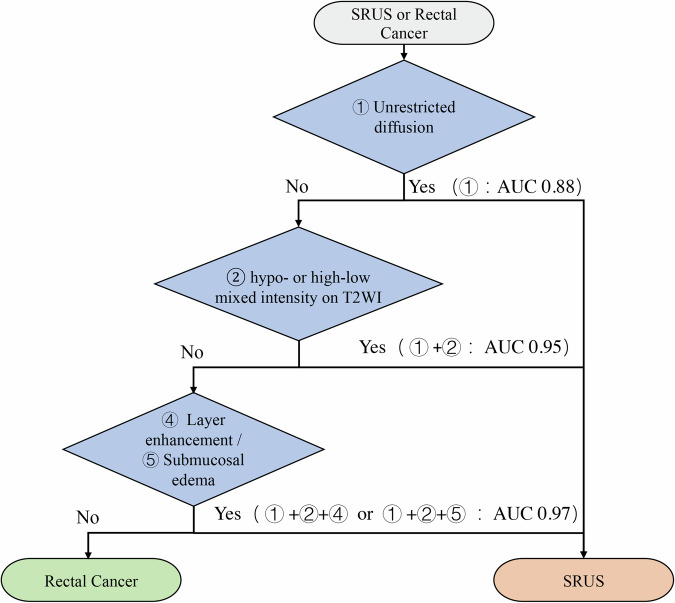
Diagnostic flowchart for differentiating SRUS from rectal cancer based on MRI findings. The three MRI features, including unrestricted diffusion, hypo- or high-low mixed intensity on T2WI, and layer enhancement or submucosal edema, were classified as SRUS if any or all of the features were present; otherwise, they were classified as rectal cancer

The combinations of three MRI features including unrestricted diffusion, hypo- or high-low mixed intensity on T2WI and layer enhancement (① + ② + ④) or unrestricted diffusion, hypo- or high-low mixed intensity on T2WI and submucosal edema (① + ② + ⑤) showed an excellent diagnostic performance in differentiating SRUS from rectal cancer, with AUC, sensitivity, specificity, positive predictive value, negative predictive value, and accuracy of 0.97 (95% CI: 0.92, 1.00), 93% (28 of 30), 100% (120 of 120), 100% (28 of 28), 98% (120 of 122), and 99% (148 of 150), respectively. In addition, the combinations of three MRI features outperformed the single MRI feature or the combinations of two MRI features in differentiating SRUS from rectal cancer (as shown in Fig. 6, Tables 4 and S4).

**Table 4:** Diagnostic performance of single MRI feature and the combinations of three MRI features for differentiating SRUS from rectal cancer

Characteristic	AUC	Sensitivity	Specificity	PPV	NPV	Accuracy
① Unrestricted diffusion	(0.88) [0.81, 0.96]	23/30 (77) [58, 90]	120/120 (100) [97, 100]	23/23 (100) [85, 100]	120/127 (94) [89, 98]	143/150 (95) [91, 98]
② Hypo- or high-low mixed intensity on T2WI	(0.88) [0.81, 0.96]	23/30 (77) [58, 90]	120/120 (100) [97, 100]	23/23 (100) [85, 100]	120/127 (94) [89, 98]	143/150 (95) [91, 98]
③ Ulceration	(0.79) [0.70, 0.88]	19/30 (63) [44, 80]	114/120 (95) [89, 98]	19/25 (76) [55, 91]	114/125 (91) [85, 96]	133/150 (89) [82, 93]
④ Layer enhancement	(0.70) [0.61, 0.79]	12/30 (40) [23, 59]	120/120 (100) [97, 100]	12/12 (100) [74, 100]	120/138 (87) [80, 92]	132/150 (88) [82, 93]
⑤ Submucosal edema	(0.68) [0.60, 0.77]	11/30 (37) [20, 56]	120/120 (100) [97, 100]	11/11 (100) [72, 100]	120/139 (86) [79, 92]	131/150 (87) [81, 92]
① + ② + ③	(0.94) [0.89, 0.99]	28/30 (93) [78, 99]	114/120 (95) [89, 98]	28/34 (82) [65, 93]	114/116 (98) [94, 100]	142/150 (95) [90, 98]
① + ② + ④	(0.97) [0.92, 1.00]	28/30 (93) [78, 99]	120/120 (100) [97, 100]	28/28 (100) [88, 100]	120/122 (98) [94, 100]	148/150 (99) [95, 100]
① + ② + ⑤	(0.97) [0.92, 1.00]	28/30 (93) [78, 99]	120/120 (100) [97, 100]	28/28 (100) [88, 100]	120/122 (98) [94, 100]	148/150 (99) [95, 100]

Data in parentheses are percentages, data in brackets are 95% confidence intervals

*AUC* area under the receiver operating characteristic curve, *PPV* positive predictive value, *NPV* negative predictive value

## Discussion

Solitary rectal ulcer syndrome (SRUS) is a very rare chronic disease. To the best of the authors’ knowledge, the literature on MRI findings in SRUS patients consists only of case reports without systematic evaluation [1, 2, 4], with the exception of a case series of 28 patients with MR defecography findings [14]. Therefore, the MRI characteristics of SRUS patients are poorly understood. In fact, it is often misdiagnosed as rectal cancer [1, 2]. This may lead to inappropriate treatment and unnecessary surgery. Therefore, an accurate diagnosis of SRUS is critical to provide appropriate treatment options. In our study, SRUS patients were significantly younger, had a lower rate of CEA levels and a longer history of symptoms than rectal cancer patients. SRUS patients had a significantly higher incidence of ulceration, submucosal edema, unrestricted diffusion, hypo- or high-low mixed intensity on T2WI, and layer enhancement compared to rectal cancer patients. Interestingly, in the combinations of three MRI features, including unrestricted diffusion, hypo- or high-low mixed intensity on T2WI, and layer enhancement or submucosal edema, showed an excellent diagnostic performance in differentiating SRUS from rectal cancer, outperforming the single MRI feature and the combinations of two MRI features.

In our study, there is a clear predominance of men among the patients with SRUS. After searching the relevant literature, we found that, although most of the studies have a slight female predominance, a few case series are the same as our finding of male predominance [15, 16]. Previous literature [1, 2, 4] cited case reports of MRI findings in SRUS patients (Table S1) and reported that they were misdiagnosed as rectal cancer. Amaechi et al [1] reported the MRI findings of an SRUS patient who was misdiagnosed with rectal tumors and suggested that SRUS lesions tended to occur in the anterior wall of the rectum. However, in our study, most of the lesions showed circumferential thickening. Although previous studies have reported that the presence of cystic lesions in the submucosa is indicative of SRUS [2]. However, we found cystic lesions in the submucosa in only one patient (1/30, 3.33%) in our study, and the pathological examination showed cystic degeneration. According to a case report by Choi et al [4], lesions showing focal thickening with ulceration can easily be misdiagnosed as rectal cancer. The same findings were confirmed in our study, and the six SRUS patients who were misdiagnosed as rectal cancer on initial MRI all showed focal thickening with bowel involvement of less than 50 mm [17]. In our study, diffusion restriction was observed in some SRUS cases (23.33%). Explaining the reason for the restricted diffusion in SRUS is a challenge that needs further study, and we can only speculate about possible mechanisms according to a study on inflamed bowel [18]. Inflammatory cell infiltration may lead to increased cell density and reduced extracellular space, which may contribute to the restricted diffusion of water molecules. We found that, in the combinations of three MRI features including unrestricted diffusion, hypo- or high-low mixed intensity on T2WI and layer enhancement or submucosal edema showed excellent diagnostic performance in differentiating SRUS from rectal cancer (AUC, 0.97). In addition, the combination of three MRI features such as unrestricted diffusion, hypo- or high-low mixed intensity on T2WI and submucosal edema (① + ② + ⑤), all obtained from the non-enhancement MRI sequences, can well discriminate SRUS and rectal cancer, which may be superior to the combination of three MRI features, such as unrestricted diffusion, hypo- or high-low mixed intensity on T2WI and layer enhancement (① + ② + ④), which need to be combined with enhanced MRI sequences. The European Society of Gastrointestinal and Abdominal Radiology (ESGAR) consensus guidelines recommend against routine IV Gd enhancement for primary rectal cancer staging, as morphological T2-weighted imaging (T2WI) and diffusion-weighted imaging (DWI) are sufficient for local staging in most cases [19]. This practice is consistent with our observation that the three key discriminative features (unrestricted diffusion, hypo- or high-low mixed intensity on T2WI, and submucosal edema) derived from non-enhancement MRI sequences achieved an AUC of 0.97, meaning that robust diagnostic performance can be demonstrated even in the absence of contrast enhancement.

In addition, biopsies remove only superficial mucosal, submucosal, or ulcerated tissue, which may not fully reflect the overall condition of the lesions. In contrast, MRI, on the other hand, with its high soft tissue resolution and multiple imaging planes, can clearly depict the overall condition of the lesions and may offer some advantages over biopsy pathology. Previous case reports [2, 4] have recommended that in cases where the diagnosis of rectal cancer is uncertain based on MRI, clinicians should perform repeated deep-forceps macrobiopsies or repeated endoscopic biopsy to exclude the possibility of malignancy. According to the studies reported in the literature [20], conservative treatment is the first-line approach to cure SRUS. For patients with moderate to high-grade rectal prolapse, rectopexy should be considered. In cases where the condition is severe and leads to visceral organ prolapse, abdominal visceral suspension may be performed. However, when rectal cancer is diagnosed, direct surgery or neoadjuvant therapy followed by surgery is generally the preferred approach [21]. This highlights the importance of an accurate diagnosis to guide clinical decision-making and selection of the appropriate treatment.

This study has several limitations. First, it is a retrospective study conducted in a single center with a relatively small sample size. Second, we relied only on biopsy pathology for some patients. Due to the influence of sampling location and depth, the pathological results may not fully reflect the overall MRI findings. Third, we only included rectal adenocarcinoma patients with ≤ T2N0 pathological staging for the differentiation analysis. The decision to include only ≤ T2N0 rectal cancers was intentional to minimize confounding factors. Advanced-stage tumors (≥ T3) often have distinct imaging findings, such as extramural invasion, lymphadenopathy or distant metastases. These findings inherently facilitate differential diagnosis in clinical practice. In contrast, early-stage rectal cancers (≤ T2N0) share overlapping morphological features with SRUS (e.g., intact muscularis propria, rare occurrence of lymphadenopathy), posing a greater diagnostic challenge. Fourth, the other rectal pathological types, such as mucinous adenocarcinoma, signet ring cell carcinoma, and other rectal conditions, such as inflammatory bowel disease, infectious proctitis, or benign ulcers, were not included in our study but warrant further investigation in the future.

## Conclusion

This study found that the combinations of three MRI features, including unrestricted diffusion, hypo- or high-low mixed intensity on T2WI, and layer enhancement or submucosal edema, showed an excellent diagnostic performance in differentiating SRUS from rectal cancer, outperforming the single MRI feature and the combinations of two MRI features.

## Supplementary information

ELECTRONIC SUPPLEMENTARY MATERIAL

## Data Availability

The datasets used and analyzed during the current study are available from the corresponding author on reasonable request by mail if necessary.

## Abbreviations

ADC: Apparent diffusion coefficient
AUC: Area under the receiver operating characteristic curve
DWI: Diffusion-weighted imaging
MRI: Magnetic resonance imaging
NPV: Negative predictive value
PPV: Positive predictive value
SRUS: Solitary rectal ulcer syndrome
SD: Standard deviation
T1WI: T1-weighted imaging
T2WI: T2-weighted imaging

## References

[1] Amaechi I, Papagrigoriadis S, Hizbullah S, Ryan SM (2010) Solitary rectal ulcer syndrome mimicking rectal neoplasm on MRI. Br J Radiol 83:e221–e224. 10.1259/bjr/24752209 20965892 10.1259/bjr/24752209 PMC3473720

[2] Blanco F, Frasson M, Flor-Lorente B, Minguez M, Esclapez P, Garcia-Granero E (2011) Solitary rectal ulcer: ultrasonographic and magnetic resonance imaging patterns mimicking rectal cancer. Eur J Gastroenterol Hepatol 23:1262–1266. 10.1097/MEG.0b013e32834b0dee 21971372 10.1097/MEG.0b013e32834b0dee

[3] Ingle SB, Patle YG, Murdeshwar HG, Hinge Ingle CR (2012) An unusual case of solitary rectal ulcer syndrome mimicking inflammatory bowel disease and malignancy. Arab J Gastroenterol 13:102. 10.1016/j.ajg.2012.02.004 22980604 10.1016/j.ajg.2012.02.004

[4] Choi YM, Song HJ, Kim MJ, Chang WY, Kim BS, Hyun CL (2016) Solitary rectal ulcer syndrome mimicking rectal cancer. Ewha Med J 39:4. 10.12771/emj.2016.39.1.28

[5] Faghih Dinevari M, Eftekharsadat A, Tarverdizadeh M et al (2023) Rectal bleeding as a symptom of solitary rectal ulcer syndrome mimicking rectal neoplasm on colonoscopy: a case report. Clin Case Rep 11:e7277. 10.1002/ccr3.7277 37113638 10.1002/ccr3.7277 PMC10126758

[6] K CS, Sharma S, Basnet B, Mishra AK (2008) Solitary rectal ulcer syndrome: uncommon cause of rectal bleeding in children. J Nepal Med Assoc 47:238–240 19079404

[7] Bonnard A, Mougenot JP, Ferkdadji L, Huot O, Aigrain Y, De Lagausie P (2003) Laparoscopic rectopexy for solitary ulcer of rectum syndrome in a child. Surg Endosc 17:1156–1157. 10.1007/s00464-002-4285-3 12728388 10.1007/s00464-002-4285-3

[8] Zhu QC, Shen RR, Qin HL, Wang Y (2014) Solitary rectal ulcer syndrome: clinical features, pathophysiology, diagnosis and treatment strategies. World J Gastroenterol 20:738–744. 10.3748/wjg.v20.i3.738 24574747 10.3748/wjg.v20.i3.738 PMC3921483

[9] Gouriou C, Siproudhis L, Chambaz M et al (2021) Solitary rectal ulcer syndrome in 102 patients: do different phenotypes make sense? Dig Liver Dis 53:190–195. 10.1016/j.dld.2020.10.041 33199231 10.1016/j.dld.2020.10.041

[10] Wessling J, Kucharzik T, Bettenworth D et al (2023) Intestinal MRI in inflammatory bowel disease—literature and survey-based recommendations regarding reporting by the German Radiological Society (DRG) and the German Competence Network for Inflammatory Bowel Diseases. Rofo 195:675–690. 10.1055/a-2036-7190 37137321 10.1055/a-2036-7190

[11] Ogawa S, Hida J, Ike H et al (2016) Selection of lymph node-positive cases based on perirectal and lateral pelvic lymph nodes using magnetic resonance imaging: study of the Japanese Society for Cancer of the Colon and Rectum. Ann Surg Oncol 23:1187–1194. 10.1245/s10434-015-5021-2 26671038 10.1245/s10434-015-5021-2

[12] Bellini D, Rivosecchi F, Panvini N et al (2019) Layered enhancement at magnetic resonance enterography in inflammatory bowel disease: a meta-analysis. World J Gastroenterol 25:4555–4566. 10.3748/wjg.v25.i31.4555 31496631 10.3748/wjg.v25.i31.4555 PMC6710183

[13] Landis JR, Koch GG (1977) The measurement of observer agreement for categorical data. Biometrics 33:159–174 843571

[14] Abdelatty MA, Halligan S, El Sayed RF, Plumb AAO (2021) Solitary rectal ulcer syndrome (SRUS): observational case series findings on MR defecography. Eur Radiol 31:8597–8605. 10.1007/s00330-021-08075-6 34357449 10.1007/s00330-021-08075-6

[15] Behera MK, Dixit VK, Shukla SK et al (2015) Solitary rectal ulcer syndrome: clinical, endoscopic, histological and anorectal manometry findings in North Indian patients. Trop Gastroenterol 36:244–250. 10.7869/tg.298 27509702 10.7869/tg.298

[16] Ejaz Z, Khan SU, Rehman RU, Jibran MS (2023) Solitary rectal ulcer syndrome in patients presenting with lower gastrointestinal bleeding: a tertiary-care hospital experience. Cureus 15:e35247. 10.7759/cureus.35247 36968942 10.7759/cureus.35247 PMC10034737

[17] Fernandes T, Oliveira MI, Castro R, Araujo B, Viamonte B, Cunha R (2014) Bowel wall thickening at CT: simplifying the diagnosis. Insights Imaging 5:195–208. 10.1007/s13244-013-0308-y 24407923 10.1007/s13244-013-0308-y PMC3999365

[18] Oto A, Zhu F, Kulkarni K, Karczmar GS, Turner JR, Rubin D (2009) Evaluation of diffusion-weighted MR imaging for detection of bowel inflammation in patients with Crohn’s disease. Acad Radiol 16:597–603. 10.1016/j.acra.2008.11.009 19282206 10.1016/j.acra.2008.11.009 PMC2721917

[19] Beets-Tan RGH, Lambregts DMJ, Maas M et al (2018) Magnetic resonance imaging for clinical management of rectal cancer: updated recommendations from the 2016 European Society of Gastrointestinal and Abdominal Radiology (ESGAR) consensus meeting. Eur Radiol 28:1465–1475. 10.1007/s00330-017-5026-2 29043428 10.1007/s00330-017-5026-2 PMC5834554

[20] Gaj F, Lai Q, Gelormini E, Ceci M, Di Saverio S, Quaresima S (2024) Efficacy of surgical treatments for the management of solitary rectal ulcer syndrome: a network meta-analysis. Colorectal Dis 26:1515–1534. 10.1111/codi.17080 38957108 10.1111/codi.17080

[21] Benson AB, Venook AP, Adam M et al (2024) NCCN guidelines® insights: rectal cancer, version 3.2024. J Natl Compr Canc Netw 22:366–375. 10.6004/jnccn.2024.0041 39151454 10.6004/jnccn.2024.0041

