# Supplementary material for: Solitary rectal ulcer syndrome: MRI findings and differentiation from rectal cancer
Source: Insights Imaging. 2025 Jun 16;16:126. doi: 10.1186/s13244-025-01979-7 (PMC12170985; doi:10.1186/s13244-025-01979-7)
Supplement: Supplementary file 1 — ELECTRONIC SUPPLEMENTARY MATERIAL [file 13244_2025_1979_MOESM1_ESM.docx]

**Solitary Rectal Ulcer Syndrome: MRI Findings and Differentiation from**

**Rectal Cancer**

**ELECTRONIC SUPPLEMENTARY MATERIAL**

**Supplement materials**

**Supplementary Table 1**

Previous public papers about imaging findings of solitary rectal ulcer syndrome **2-3**

**Supplementary Table 2**

Parameters of MR scans at our hospital **4**

**Supplementary Table 3**

Interobserver agreement of MRI features in SURS patients **5**

**Supplementary Table 4**

Diagnostic Performance of Single MRI Feature and the combinations of two MRI features for Differentiating SRUS from Rectal Cancer **6-7**

**Supplementary Fig. 1**  **8**

**Supplementary Fig. 2 9**

**Supplementary Fig. 3 10**

| **Supplementary Table 1 Previous public papers about imaging findings of solitary rectal ulcer syndrome** | | | | |
| --- | --- | --- | --- | --- |
| **Source** | **Article types** | **Number of patients** | **Imaging modalities** | **Imaging findings** |
| Abdelatty MA, et al.Solitary rectal ulcer syndrome (SRUS): observational case series findings on MR defecography. Eur Radiol 2021;31(11):8597-8605 | Article | 28 | magnetic resonance defecography (MRD) | Internal rectal intussusception, complete external rectal prolapse, anterior rectoceles, anterior and middle compartment weakness cystocele,  uterine prolapse, enterocoeles, peritoneocoele and defecatory dyssynergia. |
| Choi YM, et al. Solitary Rectal Ulcer Syndrome Mimicking Rectal Cancer. Ewha Med J 2016;39(1):4 | Case report | 1 | CT, PET/ CT and MRI | PET-CT was highly suggestive of rectal cancer (maximum standardized uptake value, SUVmax 15.4) with intense fludeoxyglucose uptake in multiple lymph nodes (SUVmax 8.1); CT/MRI showed irregular wall thickening of mid-rectum with perirectal infiltration |
| Ingle SB, et al. An unusual case of solitary rectal ulcer syndrome mimicking inflammatory bowel disease and malignancy. Arab J Gastroenterol 2012;13(2):102 | Case report | 1 | Endoscopy | Endoscopy showing a single superﬁcial ulcer with an irregular margin and edematous surrounding mucosa mimicking malignancy and IBD |
| Blanco F,et al. Solitary rectal ulcer: ultrasonographic and magnetic resonance imaging patterns mimicking rectal cancer. Eur J Gastroenterol Hepatol 2011;23(12):1262-1266 | Case report | 6 | Endorectal ultrasound and MRI | ERUS:1) uT3N1 ;2) Submucosal thickening; 3) uT2N0 ;4) uT2N0 ;5) uT3N1 ;6) uT1N1  MRI: 1) rT3N0; 2) np; 3) rT3N0 ;4) np; 5) rT3N2; 6) Not Diagnostic |
| Amaechi I, et al. Solitary rectal ulcer syndrome mimicking rectal neoplasm on MRI. Br J Radiol 2010;83(995):e221-224 | Case report | 1 | MRI | A diagnosis of ulcerating rectal lesion with provisional staging of T1N0 was made |
| Goei R,et al. Solitary rectal ulcer syndrome: findings at barium enema study and defecography. Radiology 1988;168(2):303-306 | Case report | 16 | X ray-defecography | Rectal stricture, granularity of the mucosa, and thickened rectal folds, normal by barium enema.  Intraanal rectal intussusception, intussusception was accompanied by a rectocele et al. |
| Millward SF,et al. The barium enema appearances in solitary rectal ulcer syndrome. Clin Radiol 1985;36(2):185-189. | Case report | 17 | Barium enema | Ulceration, polypoid lesion, stricture, granularity or a normal rectal mucosa may all be found on barium enema |
| Feczko PJ, et al. Solitary rectal ulcer syndrome: radiologic manifestations. AJR Am J Roentgenol 1980;135(3):499-506. | Case report | 10 | Barium enema | Rectal narrowing, rectal stricture, ulcer, polypoid mass, small polyps et.al by barium enema |

**Supplementary Table2** **Parameters of MR scans at our hospital**

| **Scanners** | **Sequence** | **FOV (mm)** | **Slice Thickness**  **(mm)** | **Slice gap** | **Matrix** | **TR (ms)** | **TE (ms)** | **NEX** |
| --- | --- | --- | --- | --- | --- | --- | --- | --- |
| **GE 3.0T**  **(Discovery MR 750)** | T2WI | 22-24 | 3 | 0.5 | 320×256 | 4038-6120 | 120 | 4-14 |
|  | DWI | 40 | 3 | 0.5 | 160×160 | 3000 | Minimum | 4 |
|  | LAVA mask | 34 | 4 | -2 | 292×256 | 5.5 | Minimum | 1 |
|  | LAVA+C | 34 | 4 | -2 | 292×256 | 5.5 | Minimum | 1 |
| **GE 1.5T (Optima MR360)** | T2WI | 22-25 | 3 | 0.5 | 288×256 | 3270-3330 | 120 | 1-4 |
|  | DWI | 40 | 3 | 0.5 | 192×192 | 2614 | Minimum | 4 |
|  | LAVA mask | 38 | 4 | -2 | 320×224 | 6.8 | 3.1 | 1 |
|  | LAVA+C | 38 | 4 | -2 | 320×224 | 6.8 | 3.1 | 1 |
| **United Imaging 1.5T (uMR S70)** | T2WI | 22-25 | 3 | 0.5 | 320×224 | 4109-5049 | 95-105 | 3 |
|  | DWI | 40 | 3 | 0.5 | 192×192 | 5295 | 96 | 4 |
|  | t1_quick3d tra_fs_mask | 38 | 4 | -2 | 320×224 | 5.5 | 2.43 | 1 |
|  | t1_quick3d_tra fs_tra+ C | 38 | 4 | -2 | 320×224 | 5.5 | 2.43 | 1 |

Notes. - FOV, field of view; TR, repetition time; TE, echo time; NEX, number of excitations; T2WI, T2-weighted imaging; DWI, diffusion-weighted imaging; LAVA, liver acquisition with volume acceleration; FOV and slice thickness are measured in millimeters. TR and TE are measured in milliseconds.

**Supplementary Table 3 Interobserver agreement of MRI features in SURS patients**

| Features | κ Values | *P* Values |
| --- | --- | --- |
| Ulceration | 0.623±0.132 | 0.000 |
| Submucosal edema | 0.851±0.101 | 0.000 |
| Cystic lesions in the submucosa | 1.000±0.000 | 0.000 |
| Enlarged mesorectal lymph nodes | 0.889±0.109 | 0.000 |
| Layer enhancement | 0.930±0.069 | 0.000 |
| Enhancement pattern | 0.870±0.127 | 0.000 |
| Restricted diffusion | 0.793±0.138 | 0.000 |
| Intensity on T2-weighted imaging | 0.845±0.076 | 0.000 |
| Muscularis propria intact | 1.000±0.000 | 0.000 |

**Supplementary Table 4 Diagnostic Performance of Single MRI Feature and the combinations of two MRI features for Differentiating SRUS from Rectal Cancer**

| **Characteristic** | **AUC** | **Sensitivity** | **Specificity** | **PPV** | **NPV** | **Accuracy** |
| --- | --- | --- | --- | --- | --- | --- |
| **① Unrestricted diffusion** | (0.88) [0.81,0.96] | 23/30  (77) [58,90] | 120/120  (100) [97,100] | 23/23  (100) [85,100] | 120/127  (94) [89,98] | 143/150  (95) [91,98] |
| **② hypo- or high-low mixed intensity on T2WI** | (0.88) [0.81,0.96] | 23/30  (77) [58,90] | 120/120  (100) [97,100] | 23/23  (100) [85,100] | 120/127  (94) [89,98] | 143/150  (95) [91,98] |
| **③ Ulceration** | (0.79) [0.70,0.88] | 19/30  (63) [44,80] | 114/120  (95) [89,98] | 19/25  (76) [55,91] | 114/125  (91) [85,96] | 133/150  (89) [82,93] |
| **④ Layer enhancement** | (0.70) [0.61,0.79] | 12/30  (40) [23,59] | 120/120  (100) [97,100] | 12/12  (100) [74,100] | 120/138  (87) [80,92] | 132/150  (88) [82,93] |
| **⑤ Submucosal edema** | (0.68) [0.60,0.77] | 11/30  (37) [20,56] | 120/120  (100) [97,100] | 11/11  (100) [72,100] | 120/139  (86) [79,92] | 131/150  (87) [81,92] |
| **① + ②** | (0.95) [0.90,1.00] | 27/30  (90) [73,98] | 120/120  (100) [97,100] | 27/27  (100) [87, 100] | 120/123  (98) [93,99] | 147/150  (98) [94,100] |
| **① + ③** | (0.93) [0.87,0.98] | 27/30  (90) [73,98] | 114/120  (95) [89,98] | 27/33  (82) [65, 93] | 114/117  (97) [93,99] | 141/150  (94) [89,97] |
| **① + ④** | (0.93) [0.87,1.00] | 26/30  (87) [69,96] | 120/120  (100) [97,100] | 26/26  (100) [87, 100] | 120/124  (97) [92,99] | 146/150  (97) [93,99] |
| **① + ⑤** | (0.93) [0.87,1.00] | 26/30  (87) [69,96] | 120/120  (100) [97,100] | 26/26  (100) [87, 100] | 120/124  (97) [92,99] | 146/150  (97) [93,99] |
| **② + ③** | (0.91) [0.84,0.97] | 26/30  (87) [69,96] | 114/120  (95) [89,98] | 26/32  (81) [64, 93] | 114/118  (97) [92,99] | 140/150  (93) [88,97] |
| **② + ④** | (0.92) [0.85,0.98] | 25/30  (83) [65,94] | 120/120  (100) [97,100] | 25/25  (100) [86, 100] | 120/125  (96) [91,99] | 145/150  (97) [92,99] |
| **② + ⑤** | (0.90) [0.83,0.97] | 24/30  (80) [61,92] | 120/120  (100) [97,100] | 24/24  (100) [86, 100] | 120/126  (95) [90,98] | 144/150  (96) [91,99] |

Note- Data in parentheses are percentages, data in brackets are 95% confidence intervals. AUC = area under the receiver operating characteristic curve, PPV = positive predictive value, NPV = negative predictive value.


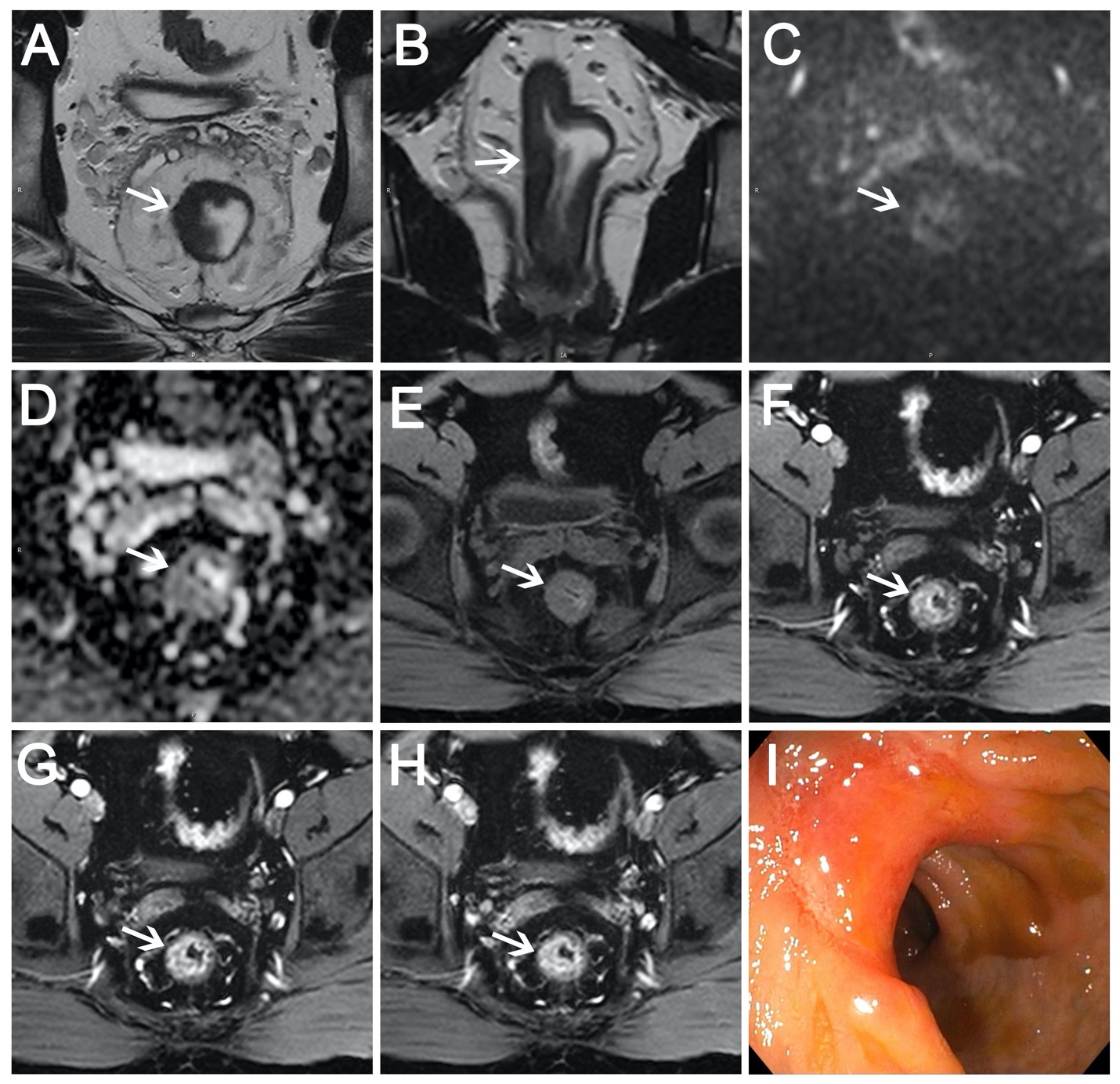


**Supplement Fig1. A 30-year-old man with a history of altered bowel habits for 36 months and rectal prolapse for 2 months.** (A-H) MRI demonstrated an area of focal thickening in the right lateral rectal wall approximately 70 mm from the anal verge. (A) Axial and (B) sagittal T2WI show hypointense signal involving a bowel length of 34 mm. (C) Unrestriction diffusion was observed on DWI and (D) ADC map. (F) T1WI shows isointense signal. (G-H) Persistent enhancement was observed on the multiphase contrast-enhanced scans. Colonoscopy (I) shows an ulcerative mass 70mm from the anal margin.


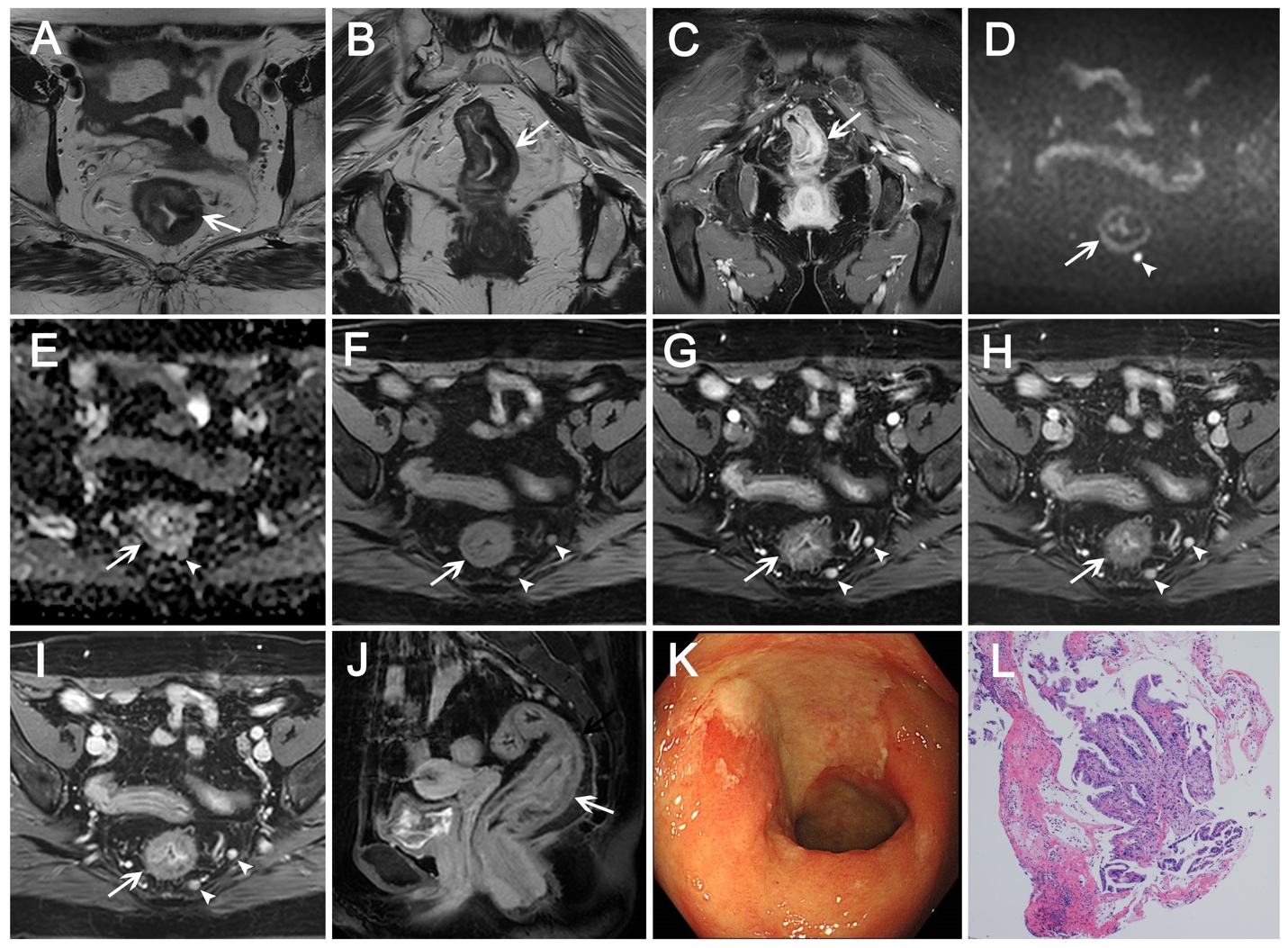


**Supplement Fig2. A 69-year-old woman with a 20-year history of recurrent intermittent constipation.** MRI showed diffuse thickening and multiple ulcerations of the middle and upper rectal segment involving approximately 76 mm of the rectal wall. (A) Axial and (B) coronal T2WI show high-low mixed intensity and unrestricted diffusion on (D) DWI and (E) ADC map. (F) T1WI shows isointense signal, and (C, G-J) contrast-enhanced scan show persistent and layer enhancement. Several slightly enlarged mesenteric lymph nodes are seen (arrowheads in D-I). (K) Colonoscopy shows ulcerations at 100mm from the anal verge with white moss covering a half of the circumference, and the affected rectal wall was hard, swollen with surrounding mucosal hyperemia. (L) Photomicrograph shows surface erosions, fibromuscular obliteration in the lamina propria, and distorted crypt architecture (hematoxylin-eosin stain, magnification, ×100).


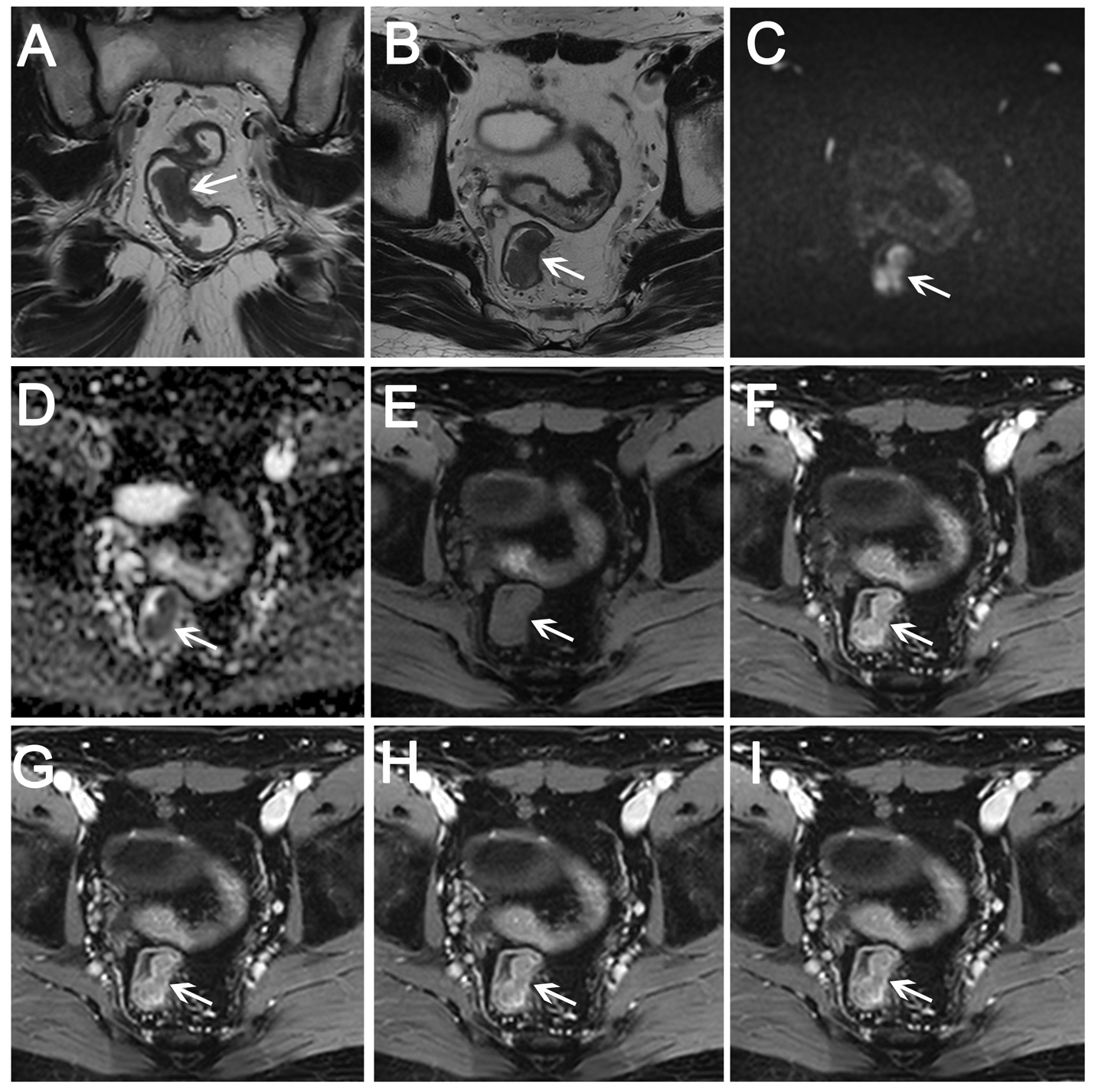


**Supplement Fig3. A 53-year-old man with a two-month history of rectal bleeding.** (A) Coronal and (B) axial T2WI show slight hyperintensity over a bowel length of 30mm. (C) DWI and (D) ADC map show significant diffusion restriction. (E) T1WI shows isointense signal. (E-I) Contrast enhanced imaging shows significant enhancement in the arterial phase and then washout in the venous phase. The patient's pathology confirmed a T2N0 stage rectal adenocarcinoma.
